# Gelsolin inhibits autophagy by regulating actin depolymerization in pancreatic ductal epithelial cells in acute pancreatitis

**DOI:** 10.1590/1414-431X2023e12279

**Published:** 2023-01-27

**Authors:** Huiying Yang, Zhihai Liang, Jinlian Xie, Qing Wu, Yingying Qin, Shiyu Zhang, Guodu Tang

**Affiliations:** 1 Department of Gastroenterology, First Affiliated Hospital of Guangxi Medical University, Nanning, China; 2 Department of Gastroenterology, Second Affiliated Hospital of Guangxi Medical University, Nanning, China

**Keywords:** Gelsolin, Autophagy, Actin filaments, Pancreatic ductal epithelial cells, Acute pancreatitis

## Abstract

Gelsolin (GSN) can sever actin filaments associated with autophagy. This study investigated how GSN-regulated actin filaments control autophagy in pancreatic ductal epithelial cells (PDECs) in acute pancreatitis (AP). AP was produced in a rat model and PDECs using caerulein (CAE). Rat pancreatic duct tissue and HPDE6-C7 cells were extracted at 6, 12, 24, and 48 h after CAE treatment. HPDE6-C7 cells in the presence of CAE were treated with cytochalasin B (CB) or silenced for *GSN* for 24 h. Pancreatic histopathology and serum amylase levels were analyzed. Cellular ultrastructure and autophagy in PDECs were observed by transmission electron microscopy after 24 h of CAE treatment. The expression of GSN and autophagy markers LC3, P62, and LAMP2 was evaluated in PDECs by immunohistochemistry and western blotting. Actin filaments were observed microscopically. Amylase levels were highest at 6 h of AP, and pancreatic tissue damage increased over time. Mitochondrial vacuolization and autophagy were observed in PDECs. CAE increased GSN expression in these cells over time, increased the LC3-II/LC3-I ratio and LAMP2 expression at 24 and 6 h of treatment, respectively, and decreased P62 expression at all time points. CB treatment for 24 h decreased the LC3-II/LC3-I ratio and LAMP2 expression, increased P62 levels, but had no impact on GSN expression in CAE-treated PDECs. CAE induced actin depolymerization, and CB potentiated this effect. *GSN* silencing increased the LC3-II/LC3-I ratio and LAMP2 expression and reduced actin depolymerization in CAE-treated PDECs. GSN may inhibit autophagosome biogenesis and autophagosome-lysosome fusion by increasing actin depolymerization in PDECs in AP.

## Introduction

The pathogenesis of acute pancreatitis (AP) is complex and multifactorial and has not been fully elucidated. Impaired autophagy, abnormal activation of trypsinogen, and the increased secretion of proinflammatory cytokines are implicated in AP (1-4).

Autophagy is a multi-step process involving autophagosome biogenesis and fusion with lysosomes, enzymatic hydrolysis of autophagosomes, and the reuse of cellular components (5). Microtubule-associated protein light chain 3 (LC3), sequestosome 1 (SQSTM1)/p62, and lysosome-associated membrane protein 2 (LAMP2) are markers of autophagic flux. During autophagy, cytosolic LC3-I is converted to LC3-II, which is incorporated into the autophagosomal membrane. The increase in the LC3-II/LC3-I ratio is a hallmark of autophagosome formation. P62 binds to LC3 and facilitates the selective degradation of protein cargo via autophagy (6). LAMP2 is a major lysosomal membrane protein involved in autophagosome-lysosome fusion (7). Autophagy is mediated by LC3-I to LC3-II conversion, increased LAMP2 protein expression, and decreased p62 protein expression (8).

Autophagy is critical in the pathogenesis of AP (9-11). Trypsinogen activation in the secretory vesicles, endosomes, lysosomes, or autophagosomes/autophagolysosomes of pancreatic acinar cells (PACs) and LC3-I to LC3-II conversion are the initiating events in AP (3). As AP progresses, autophagy impairment in PACs causes zymogen granule accumulation (1). Autophagy regulates intracellular trypsin activity in the later stage of AP (12). However, the mechanisms underlying autophagy in pancreatic ductal epithelial cells (PDECs) are not fully understood.

PDECs, one of the main components of the pancreatic ductal mucosal barrier, secrete Cl^-^ and HCO_3_^-^ and interact with PACs. Low concentrations of pancreatitis-inducing factors stimulate bicarbonate and fluid secretion by ductal cells, inhibit trypsinogen autoactivation, and remove toxic compounds/enzymes from the pancreas. However, high concentrations of pancreatitis-inducing factors reverse these effects and impair PACs and PDECs (13-16). In our previous studies, AP was mimicked in PDECs *in vitro* using CAE and led to pyroptosis and barrier function impairment, in line with the results in a rat model of AP (17-19). However, the role of autophagy in this *in vitro* model has not been determined.

Actin filaments are major cytoskeletal components that regulate autophagy via autophagosome formation, movement, and fusion with lysosomes (20,21); however, the role of the actin cytoskeleton in autophagy has been studied primarily in cancer models but not in PDECs (22). We have shown that the actin polymerization inhibitor cytochalasin B (CB) increased LC3-I to LC3-II conversion in HPDE6-C7 cells, suggesting that actin filaments were involved in autophagy in these cells (Huiying Yang, Zhihai Liang, Jinlian Xie, Qing Wu, Yingying Qin, Shiyu Zhang, Guodu Tang, unpublished data).

Gelsolin (GSN) is an actin-binding protein that regulates actin dynamics and participates in morphological, motor, and metabolic activities (23). GSN binds to and regulates actin by nucleating, capping, cross-linking, and severing actin filaments (24-26). GSN is also implicated in cellular immune response, apoptosis, and cell movement (27). Our previous study showed that CAE increased GSN protein expression and disrupted the actin filament network in HPDE6-C7 cells. *GSN* silencing reduced these effects, suggesting that GSN might depolymerize actin filaments in PDECs in AP (18). However, the role of GSN-regulated actin filaments in autophagy in PDECs in AP is not fully understood.

We hypothesize that GSN inhibits autophagy in PDECs by depolymerizing actin filaments. To test this hypothesis, AP was mimicked in PDECs using CAE, and autophagy was monitored *in vivo* and *in vitro*. Furthermore, actin depolymerization was inhibited using CB, and *GSN* was silenced in CAE-treated HPDE6-C7 cells to assess the effect of GSN-regulated actin filaments on autophagy in PDECs.

## Material and Methods

### Animal model

The study protocols were approved by the Animal Care and Use Committee of Guangxi Medical University (No. 201904002). All experiments were performed in accordance with the Guide for the Care and Use of Laboratory Animals.

Sixty-four 8-week-old specific-pathogen-free Sprague-Dawley healthy male rats weighing 230-250 g were randomly divided into a control group (CG, n=32) and an acute pancreatitis group (APG, n=32). All animals fasted for 12 h before treatment and had free access to water. The APG was intraperitoneally injected seven times with caerulein (CAE) (C9026, Sigma-Aldrich, USA) (50 μg/kg body weight, once an hour), and the CG was injected with saline. The rats were sacrificed and dissected 6, 12, 24, and 48 h after injection.

### Amylase levels and pancreatic histopathology

Serum amylase levels were determined using an automatic biochemical analyzer. Pancreatic tissue sections were stained with hematoxylin-eosin, and the severity of pancreatitis was scored by two pathologists, blinded to group allocation, according to the degree of necrosis, inflammation, hemorrhage, and edema (28).

### Immunohistochemistry (IHC) staining

Paraffin-embedded pancreatic tissue slices (4 μm) were deparaffinized with xylene, dehydrated through a graded ethanol series, subjected to heat-induced antigen retrieval using Tris-EDTA buffer pH 9.0 (G1203; Servicebio, China), and incubated with 3% hydrogen peroxide for 25 min in the dark and 5% normal goat serum (SL038; Solarbio, China) for 30 min at 37°C. Samples were incubated with anti-GSN antibody (1:500; ab74420; Abcam, UK) for 16-18 h at 4°C and horseradish peroxidase-conjugated goat anti-rabbit IgG (H+L) (1:200, GB23303; Servicebio) for 50 min at room temperature. Sections were stained with diaminobenzidine (brown) (G1212, Servicebio), counterstained with hematoxylin (blue) for 3 min, dehydrated through a graded ethanol series, mounted on coverslips, and observed under a light microscope at 400× magnification. DAB-positive cells were considered GSN-positive.

### Cell culture and treatment

The human PDEC line HPDE6-C7 (Jenniobio Biotechnology, China) was cultured in DMEM (Gibco, USA) supplemented with 10% fetal bovine serum (Lonsera, Uruguay), 1% penicillin-streptomycin mixture (Solarbio), and 1% L-glutamine (Solarbio). Cells were treated with CAE (10^-7^ mol/L) for 6, 12, 24, or 48 h and divided into five groups according to the duration of treatment: control (grown in CAE-free medium), 6, 12, 24, and 48 h. Another batch of cells was treated with CAE and CB (MB5434, Dalian Meilun, China) for 24 h and divided into four groups according to the type of treatment: CAE, CB, CAE+CB, and a control group (CG). The third batch of cells was silenced for *GSN*, treated with CAE for 24 h, and divided into six groups according to the type of transfection and treatment: control group (CG) (without lentiviral infection), CAE-treated CG (CAE), negative control group (NCG) (transfected with empty lentivirus), CAE-treated NCG (NC+CAE), *GSN* knockdown group (KD), and a CAE-treated KD group (KD+CAE).

### RNA interference-mediated silencing of *GSN*

Three pairs of short hairpin RNAs were designed and synthesized according to *GSN* CDS sequences and then reverse transcribed to DNA. The DNA sequences were cloned into the pcDNA6.2-GW/EmGFP-miR plasmid vector (R&S Biotechnology, China). The cloned fragments were amplified by PCR and subcloned into the pLenti6.3/V5-DEST vector (R&S Biotechnology). The lentivirus vector plasmid and lentiviral packaging mix (Invitrogen, Thermo Fisher Scientific, Inc., USA) were transiently co-transfected into 293T cells (R&S Biotechnology). Recombinant lentivirus-infected HPDE6-C7 cells with the highest degree of silencing were screened by qRT-PCR (18).

### Transmission electron microscopy (TEM)

Rat pancreatic duct tissue (1 mm^3^) and HPDE6-C7 cells were fixed with 3% glutaraldehyde for 2.5 h and 1% osmium for 2 h at 4°C, dehydrated through a graded ethanol series, and embedded in epoxy resin. Samples were cut into 70-nm sections and stained with 3% uranium acetate-lead citrate. Cellular ultrastructure and autophagy were observed by TEM.

### Western blotting

Proteins from pancreatic tissues and cells were lysed using cell lysis buffer (RIPA:PMSF, 100:1) for 30 min on ice. Protein concentration was determined using a BCA Protein Assay Kit (P0012, Beyotime, China). Proteins were separated by SDS-PAGE and electrotransferred onto polyvinylidene fluoride membranes. Membranes were blocked with 5% non-fat milk in TBST buffer for 1 h, incubated with antibodies against LC3B (ab48394, Abcam, 1:1000), SQSTM1/P62 (ab109012, Abcam, 1:10000), LAMP2 (ab199946, Abcam, 1:1000), gelsolin (ab74420, Abcam, 1:1000), and GAPDH (ab181602, Abcam, 1:10,000) overnight for 16-18 h at 4°C, and then incubated with DyLight 680-conjugated secondary anti-rabbit antibody (5366, Cell Signaling Technology, 1:10,000) for 1 h at room temperature. Immunoreactive bands were imaged using an Odyssey infrared imaging system (LI-COR, USA) and quantified by densitometry.

### Cell viability

Cell viability was determined using the CCK-8 Assay Kit (CA1210, Solarbio). HPDE6-C7 cells were grown in a 96-well plate (10^4^ cells in 100 μL of growth medium per well) for 24 h. Different concentrations of CB and 2% DMSO (10 μL per well) were added to the experimental and control wells, respectively. After that, 10 μL of CCK-8 solution was added to each well. The cells were incubated for 1 h at 37°C in a humidified 5% CO_2_ atmosphere, and absorbance was measured at 450 nm using a microplate reader.

### Flow cytometry

HPDE6-C7 cells were grown in a six-well plate and then treated with CB (0.125, 0.25, 0.5, 1.0, and 2.0 μg/mL) for 24 h. Cell apoptosis was determined using the annexin V-FITC/propidium Iodide apoptosis kit (AT101, Multi Sciences [Lianke] Biotech Co., Ltd., China), in accordance with the manufacturer's instructions. Cell apoptosis was analyzed by flow cytometry (BD Biosciences, USA).

### Actin filament staining

TRITC-phalloidin (Solarbio) was diluted to 100 nM in PBS. Cells were fixed in 4% paraformaldehyde for 15 min, permeabilized in 0.1% Triton X-100 for 5 min, blocked with 2% bovine serum albumin (Solarbio) for 20 min, stained with TRITC-phalloidin (50 μL per well) for 30 min, and mounted with anti-fading medium containing DAPI (S2100, Solarbio) in the dark. Actin filaments were imaged using an inverted fluorescence microscope (Olympus, Japan) at 530-550 nm, and cell nuclei were imaged at 460-495 nm.

### Statistical analysis

Data are reported as means±SD. Differences between groups were analyzed using one-way analysis of variance (ANOVA) followed by Tukey's test. Statistical analysis was performed using SPSS Statistics version 22.0 (IBM, USA) and GraphPad Prism version 5.0 (USA). P-values smaller than 0.05 were considered statistically significant.

## Results

### Changes in ultrastructure, autophagy, and GSN expression in rat PDECs in AP

**Figure 1:**
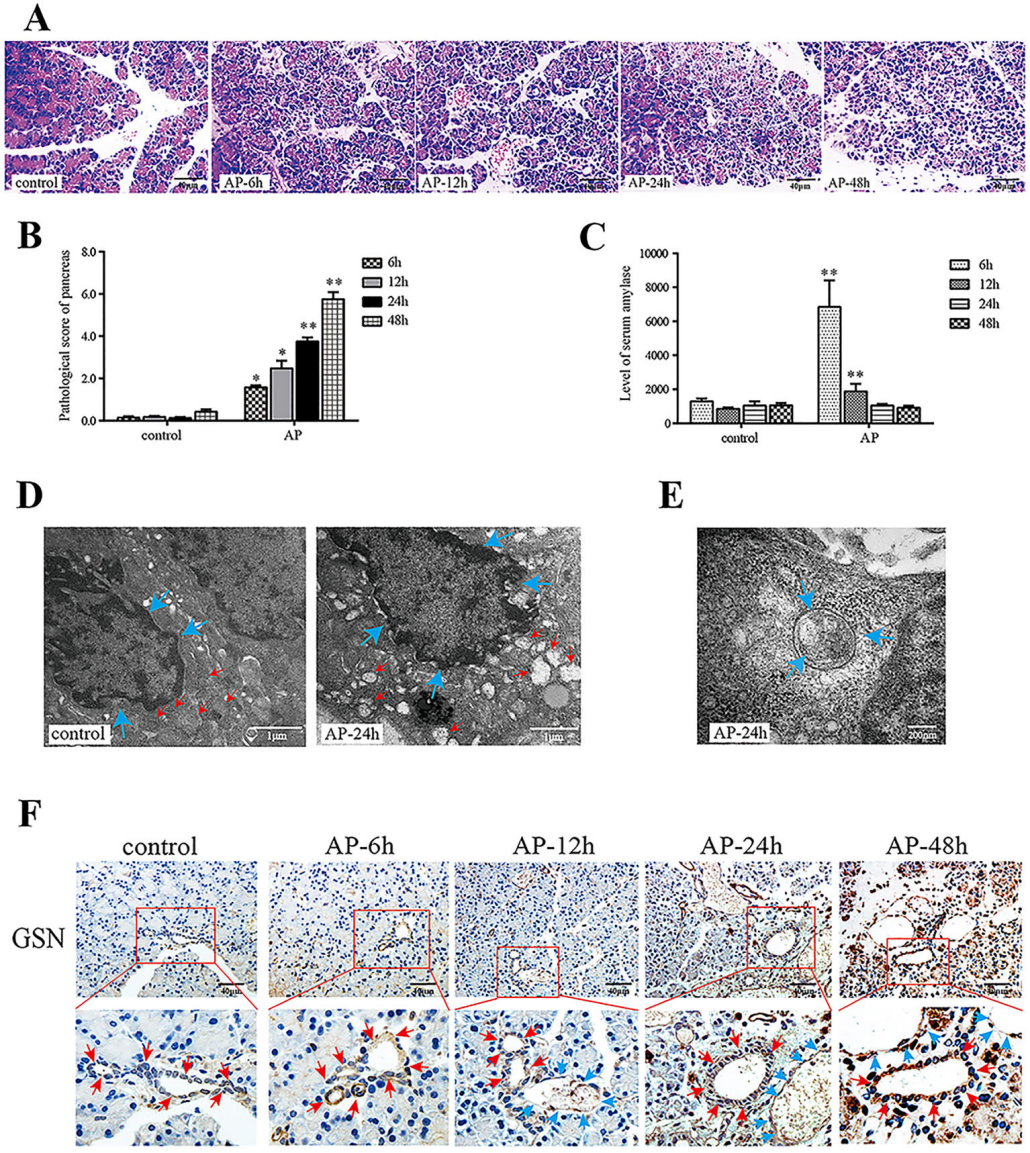
Changes in ultrastructure, autophagy, and gelsolin (GSN) expression in pancreatic duct epithelial cells (PDECs) in a rat model of acute pancreatitis (AP). The rats were injected with caerulein (CAE) (50 μg/kg body weight) for 6, 12, 24, and 48 h. **A**, Pathological changes in pancreatic tissue (hematoxylin-eosin staining, ×400, scale bar 40 μm). **B**, Pathological scores in pancreatic tissue based on the degree of necrosis, inflammation, hemorrhage, and edema. **C**, Serum amylase levels (U/L). **D**, Morphological changes in the mitochondria and nuclei of PDECs after CAE treatment for 24 h by transmission electron microscopy (×30,000, scale bar 1 μm; the red and blue arrows indicate mitochondria and nuclei, respectively). **E**, Transmission electron microscopy showing an autophagosome (blue arrows) in a PDEC after CAE treatment for 24 h (×80,000, scale bar 200 nm). **F**, Immunohistochemical expression of GSN in PDECs (×400, scale bar 40 μm). The red arrows indicate columnar PDECs of the pancreatic duct, and the blue arrows indicate flat vascular endothelial cells of blood vessels. The experiments were repeated at least three times. Data are reported as means±SD. *P<0.05, **P<0.01 *vs* the control group (one-way ANOVA followed by Tukey's test).

CAE-induced AP in rats was evaluated according to serum amylase levels and pathological changes and scores in the pancreas. The results showed that pathological scores increased because of the increase in inflammatory cell infiltration, tissue damage, and interlobular space over time (Figure 1A and B). Amylase levels were highest at 6 h of AP (Figure 1C). TEM revealed that nuclei were hyperchromatic, deformed, and condensed (blue arrows), and mitochondria were swollen and vacuolated (red arrows) (Figure 1D). Autophagosomes were observed at 24 h of AP (Figure 1E). Immunohistochemistry showed that GSN protein was expressed in PDECs and PACs (Figure 1F).

### Changes in ultrastructure, autophagy, GSN expression, and actin dynamics in CAE-treated HPDE6-C7 cells

**Figure 2:**
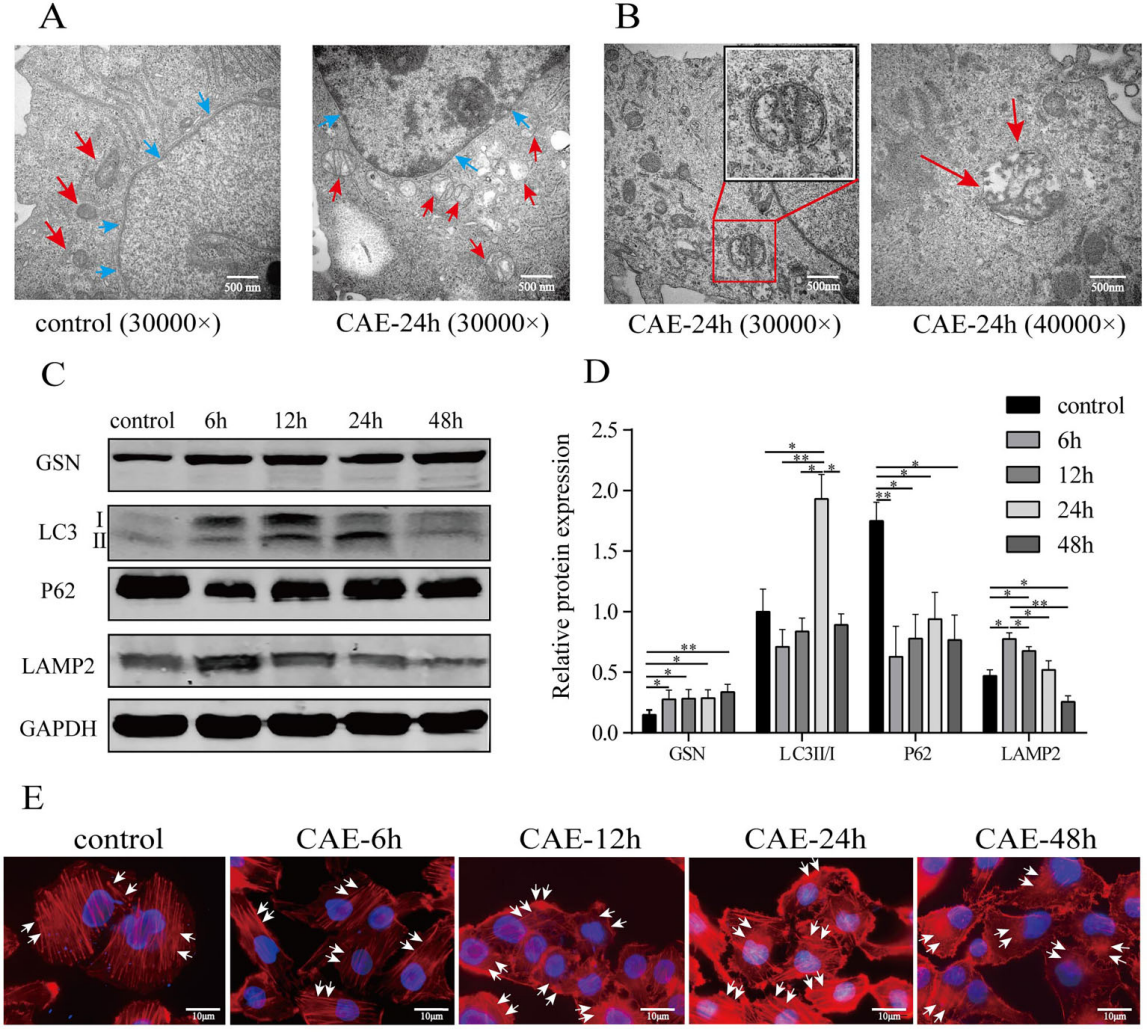
Changes in autophagy, actin dynamics, and gelsolin (GSN) expression in caerulein (CAE)-treated HPDE6-C7 cells. **A**, Ultrastructural changes in the mitochondria and nuclei of HPDE6-C7 cells treated with CAE for 24 h (×30,000, scale bar 500 nm). The red and blue arrows indicate mitochondria and nuclei, respectively. **B**, Autophagy in HPDE6-C7 cells treated with CAE for 24 h (left panel, ×30,000; right panel, ×40,000, scale bar 500 nm). The red box shows an autophagosome, and the red arrows show an autophagolysosome. **C** and **D**, Western blot and semiquantitative analyses of the expression of LC3, P62, LAMP2, and GSN in HPDE6-C7 cells treated with CAE for 6, 12, 24, and 48 h. **E**, Fluorescence microscopy analysis of changes in actin dynamics in HPDE6-C7 cells treated with CAE for 6, 12, 24, and 48 h (×1,000, scale bar 10 μm). The white arrows indicate actin filaments. The experiments were repeated at least three times. Data are reported as means±SD. *P<0.05, **P<0.01 (one-way ANOVA followed by Tukey's test).

TEM showed that nuclei were hyperchromatic, deformed, and condensed (blue arrows), and mitochondria were swollen and vacuolated (red arrows) in HPDE6-C7 cells (Figure 2A). Autophagosomes were observed at 24 h of CAE treatment (Figure 2B), consistent with the results in rat PDECs. Western blot analysis showed that CAE increased the protein expression of GSN over time (Figure 2C and D), complementary to the qualitative results in rat PDECs. In addition, the LC3-II/LC3-I ratio peaked at 24 h, and P62 expression decreased markedly at 6 h (Figure 2C and D), suggesting that autophagy was activated within 24 h of CAE treatment. LAMP2 expression peaked at 6 h, returned to control levels at 24 h, and continued to decrease at 48 h (Figure 2C and D), suggesting that autophagosome-lysosome fusion peaked within 6 h and was blocked after 24 h. Therefore, at 24 h of treatment, autophagosomes were formed, but autophagosome-lysosome fusion was inhibited, and this time point was used in subsequent experiments *in vitro*. Fluorescence microscopy showed that the reticulate pattern of actin filaments changed over time, and the staining of basolateral membranes increased (white arrows), suggesting that CAE promoted actin filament depolymerization and translocation to the basolateral membrane in a time-dependent manner (Figure 2E).

### Effects of CB on apoptosis and cell viability in CAE-treated HPDE6-C7 cells

**Figure 3:**
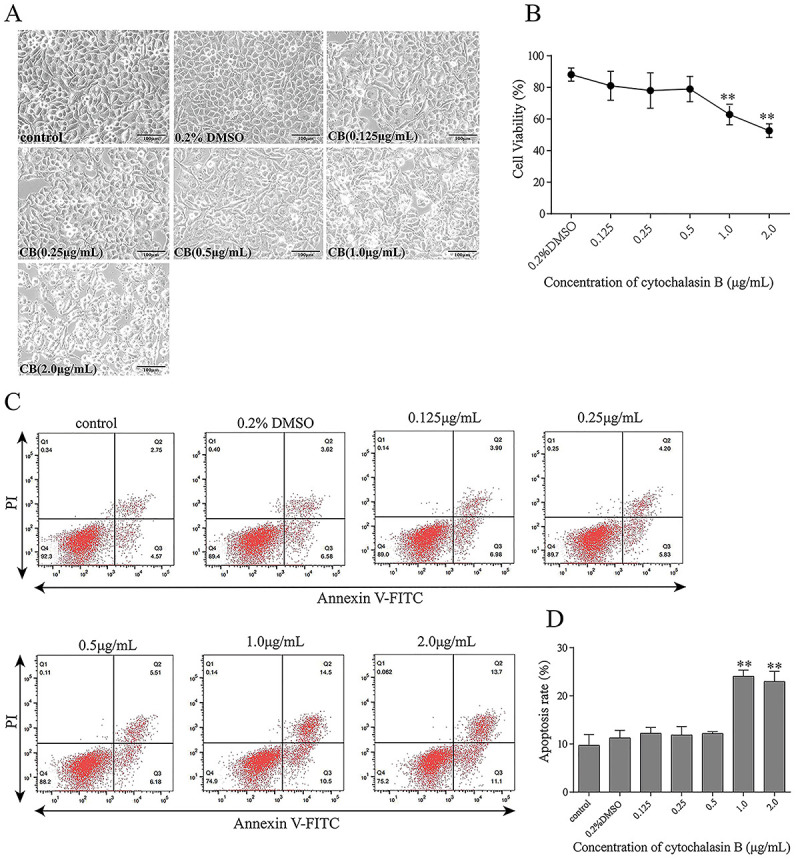
Effects of cytochalasin B (CB) on the viability and rate of apoptosis of HPDE6-C7 cells. HPDE6-C7 cells were treated with CB (0.125, 0.25, 0.5, 1.0, and 2.0 μg/mL) for 24 h. **A**, Light microscopy analysis of cell morphology (×200, scale bar 100 μm). **B**, Cell viability analysis using the CCK-8 assay. **C** and **D**, Cell apoptosis analysis by flow cytometry. The experiments were repeated at least three times. Data are reported as means±SD. **P<0.01 *vs* the control group (one-way ANOVA followed by Tukey's test).

The results of light microscopy, CCK-8 assay, and flow cytometry showed that higher concentrations of CB (1.0 and 2.0 μg/mL) changed cell morphology, decreased cell viability, and increased apoptosis (Figure 3). Therefore, the maximum non-toxic concentration of CB (0.5 μg/mL) was used in subsequent experiments.

### Effects of CB on GSN protein expression, actin dynamics, and autophagy in CAE-treated HPDE6-C7 cells

**Figure 4:**
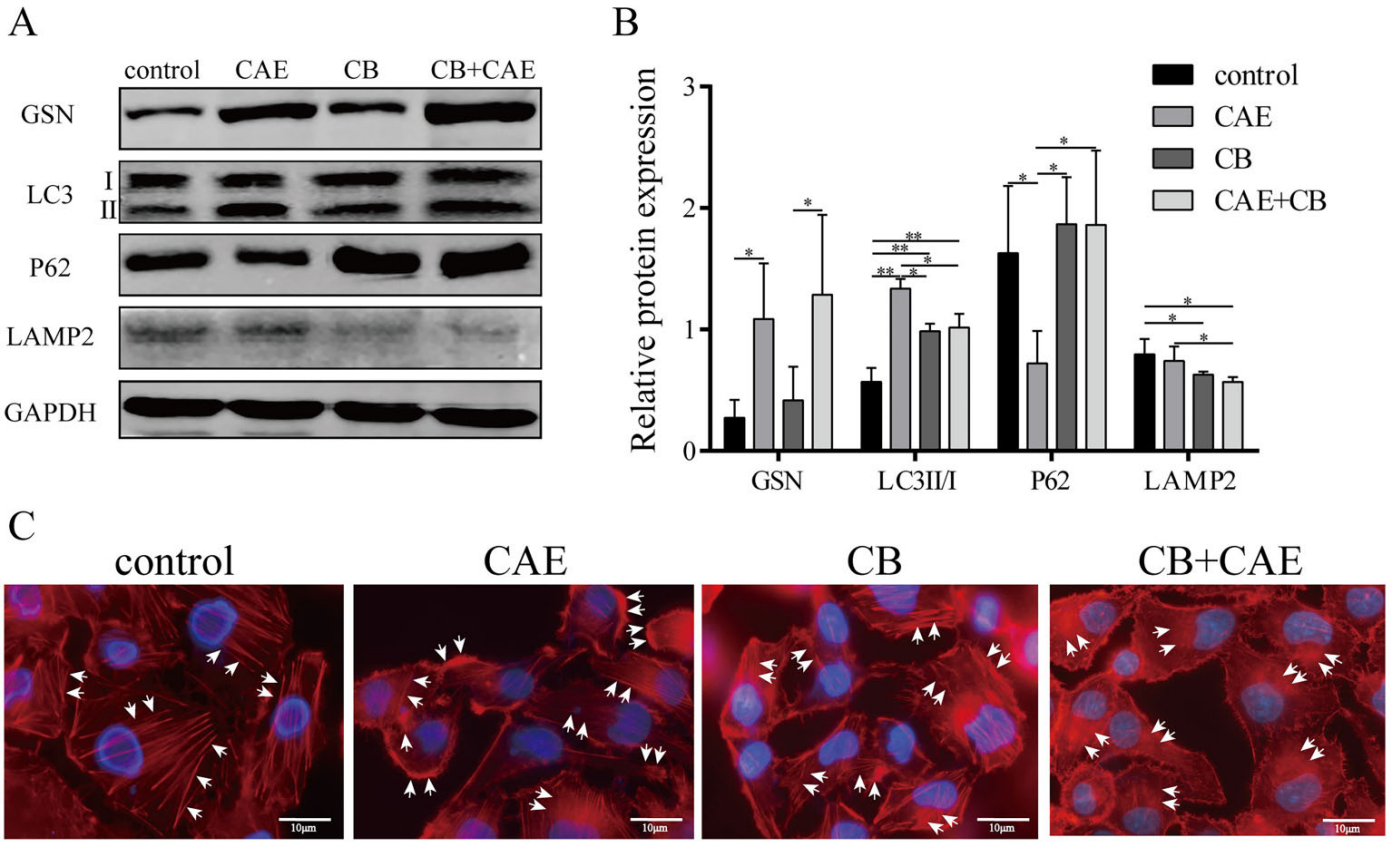
Effect of cytochalasin B (CB) on gelsolin (GSN) protein expression, actin dynamics, and autophagy in HPDE6C7 cells. HPDE6-C7 cells were treated with CB (0.5 μg/mL) and caerulein (CAE; 10^-7^ mol/L) for 24 h. **A** and **B**, Western blot and semiquantitative analysis of the expression of LC3, P62, LAMP2, and GSN. **C**, Fluorescence microscopy analysis of actin dynamics (×1,0000, scale bar 10 μm). The white arrows indicate actin filaments. The experiments were repeated at least three times. Data are reported as means±SD. *P<0.05, **P<0.01 (one-way ANOVA followed by Tukey's test).

Actin polymerization inhibitor CB was used to investigate the effects of actin depolymerization on GSN protein expression and autophagy in CAE-treated cells. Western blots showed that CAE increased GSN expression, whereas CB had no significant impact on GSN expression (Figure 4A and B). Moreover, CAE increased the LC3-II/LC3-I ratio, decreased P62 expression, but had no significant impact on LAMP2 expression at 24 h of CAE treatment, consistent with the temporal analysis of autophagy (Figure 2C and D) and demonstrating that CAE promoted autophagosome formation at 24 h. In turn, CB decreased the LC3-II / LC3-I ratio and LAMP2 expression and increased P62 expression in CAE-treated cells (Figure 4A and B), demonstrating that CB inhibited the effect of CAE on autophagosome formation and autophagosome-lysosome fusion. Fluorescence microscopy showed that CB potentiated the effect of CAE on actin filament depolymerization and translocation to the basolateral membrane (white arrows) (Figure 4C).

### Effects of GSN silencing on actin dynamics and autophagy in CAE-treated HPDE6-C7 cells

**Figure 5:**
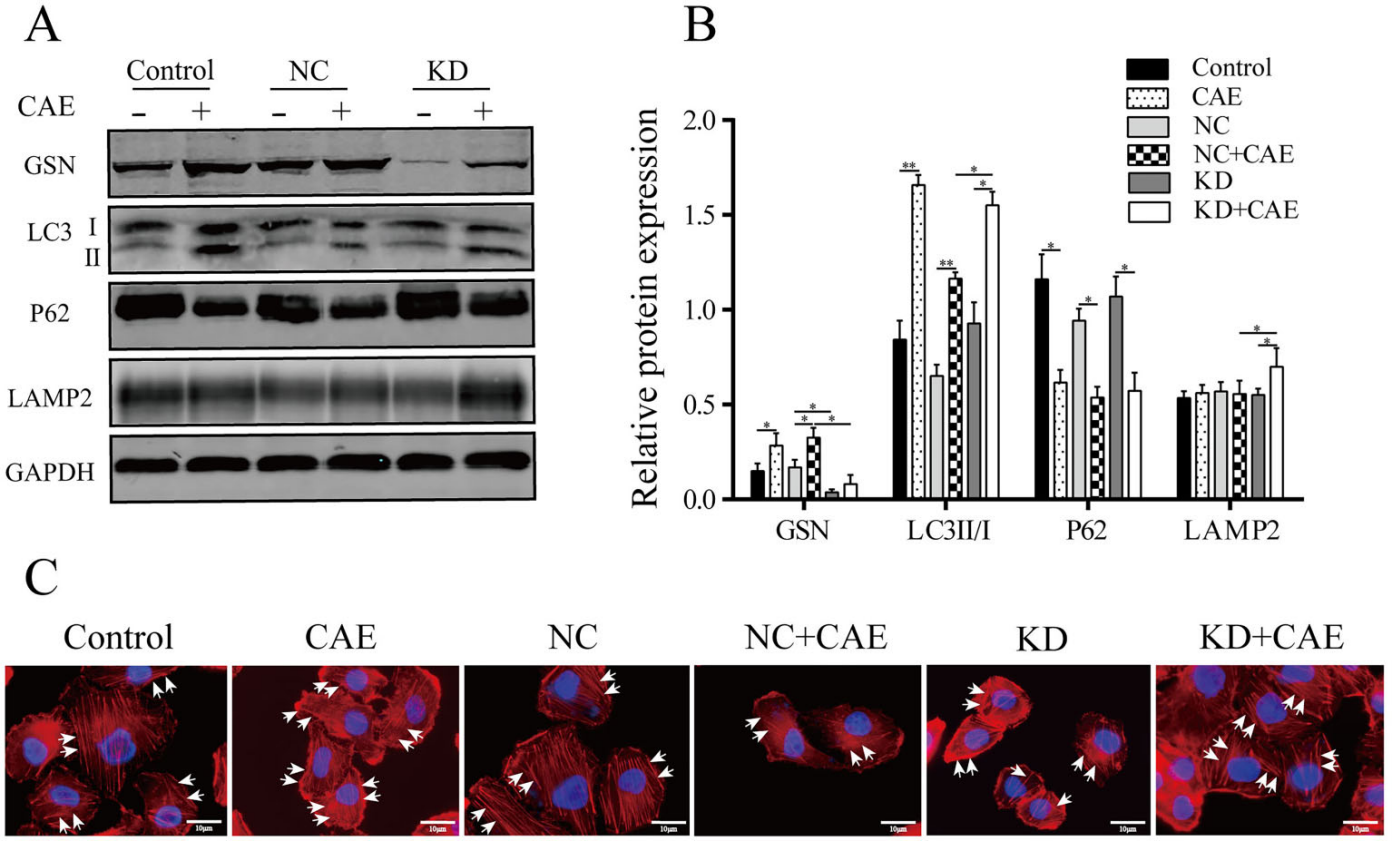
Effect of RNA interference-mediated silencing of gelsolin (*GSN*) on actin dynamics and autophagy in HPDE6-C7 cells. *GSN*-silenced HPDE6-C7 cells were treated with caerulein (CAE) for 24 h. **A** and **B**, Western blot and semiquantitative analysis of the protein expression of LC3, P62, LAMP2, and GSN. **C**, Fluorescence microscopy analysis of actin filaments (×1,000, scale bar 10 μm). The white arrows iindicate actin filaments. The experiments were repeated at least three times. Data are reported as means±SD. *P<0.05, **P<0.01 (one-way ANOVA followed by Tukey's test). Control group (without lentiviral infection); CAE: CAE-treated Control; NC: negative control group (infected with empty lentivirus); NC+CAE: CAE-treated NC; KD: *GSN* knockdown group; KD+CAE: CAE-treated KD.

Western blots showed that *GSN* silencing decreased GSN protein expression, increased the LC3-II to LC3-I ratio and LAMP2 expression, but had no significant impact on P62 expression in CAE-treated cells (Figure 5A and B), suggesting that *GSN* silencing promoted autophagosome biogenesis and autophagosome-lysosome fusion in these cells. Moreover, fluorescence microscopy showed that *GSN* knockdown reduced the effects of CAE on actin depolymerization and translocation to the basolateral membrane (Figure 5C).

## Discussion

Autophagy is involved in the pathogenesis of AP (29,30), and actin filaments play a crucial role in autophagy (20,31). Nonetheless, whether GSN, an actin filament-capping protein (25,26), affects autophagy in PDECs is unknown. This study is the first to investigate the relationship between GSN, actin dynamics, and autophagy in PDECs. The results showed that AP increased actin depolymerization, impaired autophagy, and increased GSN expression in PDECs. Moreover, GSN inhibited autophagosome formation and autophagosome-lysosome fusion in PDECs in the early stage of AP by regulating actin dynamics.

In this study, a rat model of AP was induced by CAE. AP was associated with higher pancreatic pathological scores and amylase levels. CAE increased mitochondrial vacuolization, autophagosome formation, and GSN expression in rat PDECs, consistent with the findings in CAE-treated HPDE6-C7 cells, indicating mitochondrial damage, autophagy, and GSN activation in PDECs in AP. Furthermore, the temporal analysis of the expression of autophagy markers in CAE-treated HPDE6-C7 cells revealed that autophagy was activated within 24 h of AP and autophagosome-lysosome fusion peaked within 6 h but was blocked after 24 h. In addition, actin depolymerization increased over time in these cells.

Actin filaments regulate autophagy (21) and are severed by GSN in a calcium-dependent manner (23,26,32,33). Nonetheless, the role of the actin cytoskeleton in autophagy in PDECs in AP is incompletely understood. CB inhibits autophagy by depolymerizing actin filaments (34,35). Consistent with this finding, the results of the expression of GSN and autophagy markers and actin depolymerization in CAE+CB-treated cells demonstrated that CB inhibited autophagosome biogenesis and autophagosome-lysosome fusion by increasing actin depolymerization in PDECs but had no significant effect on GSN expression at 24 h of AP.

To further investigate the effect of GSN on the actin network and autophagy in PDECs at this time point, *GSN* was silenced, and autophagy markers and actin filament dynamics were analyzed. The results showed that *GSN* silencing increased the LC3-II to LC3-I ratio and the protein expression of LAMP2 but did not significantly affect the protein expression of P62, suggesting that *GSN* knockdown promoted autophagosome and autophagolysosome biogenesis in PDECs at 24 h of AP. Moreover, *GSN* silencing reduced actin depolymerization in CAE-treated cells.

Actin filaments participate in autophagosome formation and movement and autophagosome-lysosome fusion (36-39). Consistent with these data, our results revealed that actin depolymerization using CB decreased autophagy but had no significant effect on GSN expression in PDECs at 24 h of AP.

Our findings indicated that GSN affected actin dynamics and autophagy, whereas actin dynamics affected autophagy but did not change GSN expression in PDECs at 24 h of CAE treatment, suggesting that GSN controlled autophagy by actin dynamics in PDECs at 24 h of AP. However, additional studies are warranted to elucidate temporal changes in GSN levels, actin dynamics, and autophagy in cell culture models of AP.

This study had limitations. First, autophagic flux in AP was not assessed using an inhibitor of autophagosome-lysosome fusion (40). Second, the effects of GSN overexpression on autophagic flux were not assessed.

In conclusion, active GSN inhibits autophagy in PDECs in the early stage of AP by increasing actin depolymerization. Nonetheless, additional studies are necessary to assess whether GSN is involved in other signaling pathways or interacts with other actin regulators.
